# Construct design, production, and characterization of *Plasmodium falciparum* 48/45 R0.6C subunit protein produced in *Lactococcus lactis* as candidate vaccine

**DOI:** 10.1186/s12934-017-0710-0

**Published:** 2017-05-31

**Authors:** Susheel K. Singh, Will Roeffen, Ulrik H. Mistarz, Bishwanath Kumar Chourasia, Fen Yang, Kasper D. Rand, Robert W. Sauerwein, Michael Theisen

**Affiliations:** 1 Department for Congenital Disorders, Statens Serum Institut, Artillerivej 5, 2300 Copenhagen, Denmark; 2 Department of International Health, Immunology and Microbiology, Centre for Medical Parasitology, University of Copenhagen, Copenhagen, Denmark; 3 Department of Infectious Diseases, Copenhagen University Hospital, Rigshospitalet, Copenhagen, Denmark; 4 Department of Medical Microbiology, Radboud University Nijmegen Medical Center, Nijmegen, The Netherlands; 5 Department of Pharmacy, University of Copenhagen, Copenhagen, Denmark

**Keywords:** *Plasmodium falciparum*, Malaria, Transmission blocking, *Pf*s48/45, Batch fermentation and *Lactococcus lactis*

## Abstract

**Background:**

The sexual stages of *Plasmodium falciparum* are responsible for the spread of the parasite in malaria endemic areas. The cysteine-rich *Pf*s48/45 protein, exposed on the surface of sexual stages, is one of the most advanced antigens for inclusion into a vaccine that will block transmission. However, clinical *Pf*s48/45 sub-unit vaccine development has been hampered by the inability to produce high yields of recombinant protein as the native structure is required for the induction of functional transmission-blocking (TB) antibodies. We have investigated a downstream purification process of a sub-unit (R0.6C) fragment representing the C-terminal 6-Cys domain of *Pf*s48/45 (6C) genetically fused to the R0 region (R0) of asexual stage Glutamate Rich Protein expressed in *Lactococcus lactis*.

**Results:**

A series of R0.6C fusion proteins containing features, which aim to increase expression levels or to facilitate protein purification, were evaluated at small scale. None of these modifications affected the overall yield of recombinant protein. Consequently, R0.6C with a C-terminal his tag was used for upstream and downstream process development. A simple work-flow was developed consisting of batch fermentation followed by two purification steps. As such, the recombinant protein was purified to homogeneity. The composition of the final product was verified by HPLC, mass spectrometry, SDS-PAGE and Western blotting with conformation dependent antibodies against *Pf*s48/45. The recombinant protein induced high levels of functional TB antibodies in rats.

**Conclusions:**

The established production and purification process of the R0.6C fusion protein provide a strong basis for further clinical development of this candidate transmission blocking malaria vaccine.

**Electronic supplementary material:**

The online version of this article (doi:10.1186/s12934-017-0710-0) contains supplementary material, which is available to authorized users.

## Background

The transmission of *Plasmodium falciparum* malaria from one person to another requires the production of male and female gametocytes in the human host that can be taken up by blood-feeding mosquitoes. Recent studies have indicated that a large proportion of individuals in malaria endemic areas carry gametocytes and may contribute to malaria transmission [1]. Control strategies that confer prolonged protection including vaccines, are therefore needed to effectively block malaria transmission at the population level [2]. *P. falciparum Pf*s48/45 is one of the leading transmission blocking vaccine (TBV) candidates that has entered the pipeline of clinical development (for a review see [3]). This protein is relatively cysteine-rich with multiple disulfide bonds that result in antibody epitopes which are dependent on properly folded tertiary structures rather than linear amino acid sequences. Functional in vitro studies have identified the C-terminal portion of *Pf*s48/45 (6C) containing three disulfide bonds as major target of transmission blocking (TB) antibodies [4]. This *Pf*s48/45 region is targeted by a monoclonal antibody (mAb) mAb45.1 which promotes strong TB activity in the standard membrane feeding assay (SMFA), the gold standard for assessing transmission blockade ex vivo [5–8].

The production of *Pf*s48/45 in bacterial and eukaryotic expression systems has been problematic due to insufficient protein folding capabilities of these systems. Proper folding of cysteine-rich *Pf*s48/45 depends on correct formation of disulfide bridges. Since eukaryotic cells possess a sophisticated machinery for disulfide bond formation, recombinant *Pf*s48/45 has been produced in a range of Baculovirus (*Spodoptera frugiperda* Sf9) cells [9], *Vaccinia virus* [10], *Saccharomyces cerevisiae* [11], and *Pichia pastoris* [11], *Chlamydomonas reinhardtii* [12], and *Nicotiana benthamiana* [13]. However, protein yields were rather low and in those cases where expression was successful, there was limited reactivity with mAbs against conformational TB epitopes suggesting misfolding. The bacterial *Lactococcus lactis* expression system represents a significant advancement in the production of recombinant *Pf*s48/45 [14, 15]. *L. lactis* cell factories generally recognized as safe (GRAS status) are well suited for the production of heterologous proteins and used for a wealth of food applications. In the recent years, *L. lactis* has also been used in modern biotechnology within the fields of mucosal delivery [16] generation of self-adjuvanting bacterium-like particles [17] and recombinant proteins (reviewed in [18]. *L. lactis* do not produce endotoxins or extracellular proteases. Moreover, gene expression can be controlled by a set of tightly regulated promoters in a simple and scalable fermentation process from a few ml up to thousands of liters. Recombinant proteins can be secreted into the culture medium in the absence of spore formation which clearly facilitates downstream processing. Accordingly, *L. lactis* has been used for the manufacturing of the GMZ2 malaria vaccine candidate [19–22].

To advance development of a protein-vaccine based on *Pf*s48/45, we established a manufacturing process for R0.6C in *L. lactis*. The product was characterized by mass spectrometry (MS)-based and by HPLC-based methods and tested for functional immunogenicity in rats. The specificity of induced antibodies was assessed by ELISA, and functional capacity for transmission blockade in the standard membrane-feeding assay (SMFA).

## Results

### Molecular design and expression of chimeric GLURP-*Pf*s48/45 fusion proteins

**Fig. 1:**
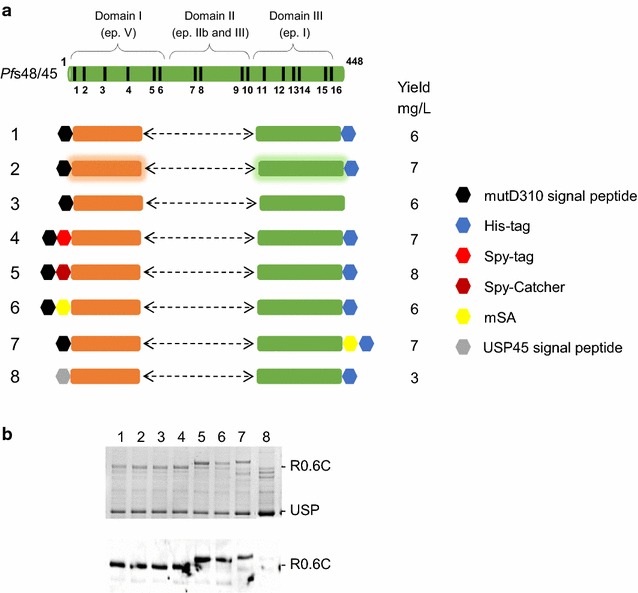
Constructs for expression of R0.6C in *L. lactis*. **a** Schematic representation of *Pf*s48/45 and the R0.6C constructs: *1* C-terminal 6xHis-tag, *2* codon optimized genes (hiligheted with* color shades*), *3* no tag, *4* N-terminal Spytag, *5* N-termianl SpyCatcher, *6* N-terminal mSA, *7* C-terminal mSA, *8* USP45 signal peptide. The protein yield of each construct was determined by densitometry scanning of the Coomassie stained SDS-PAGE gel shown below. **b** *Upper panel* Coomassie blue-stained 4–12.5% polyacrylamide gel for the constructs shown in **a**. *Lower panel* an immunoblot analysis of the same gel shown in the *upper panel* using mAb45.1

We designed a set of fusion proteins (Fig. 1a) consisting of the *Pf*s48/45 6-Cys domain to address whether expression levels of secreted R0.6C was affected by: (1) codon optimization, (2) different signal peptides, and (3) the presence and/or position of a range of affinity-tags. All constructs were transformed into *L. lactis* MG1363 and grown in 5 ml of LAB medium at 30 °C without shaking. Firstly, the codon optimized construct generated the same amount of recombinant R0.6C fusion protein as did the non-optimized construct (Fig. 1b, compare lanes 1 and 2). Secondly, we found that protein yields were similar between constructs with and without a His-tag (Fig. 1b, compare lanes 1 and 3), suggesting that the His-tag per see does not affect production yields of R0.6C. Thirdly, fusion proteins containing tags that can be used for various conjugation strategies including the SpyTag-spyCatcher technology [23, 24] and Streptavidin-mSA mediated conjugation to bacterial outer membrane vesicles [25] were explored. The addition of these tags to the N- or C-terminal end of R0.6C did not affect over all expression levels (Fig. 1b, lanes 5, 6, and 7). Finally, we showed that a native USP45 signal peptide derived from an abundantly secreted *L. lactis* protein did not increase protein yields in culture supernatants (Fig. 1b, lanes 1 and 8).

### Production of recombinant R0.6C in bioreactor

**Fig. 2:**
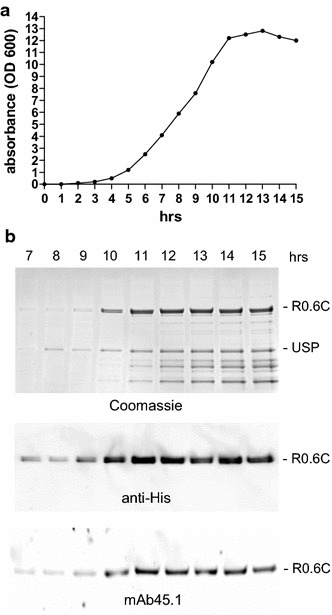
Time course analysis of the expression of R0.6C in *L. lactis*. **a** Optical density measurements at 600 nm from 0 to 15 h during expression of R0.6C. **b** Samples collected from 9 to 15 h were analyzed. For Coomassie (20 µl) and WB (10 µl) samples without a reducing agent were loaded on SDS-PAGE gel. *Upper panel* Coomassie blue-stained 4–12.5% polyacrylamide gel. *Middle panel* an WB analysis of the same gel shown in the *upper panel* using anti-His antibody. *Lower panel* an immune blot analysis of the same gel shown in the *upper panel* using mAb45.1

**Fig. 3:**
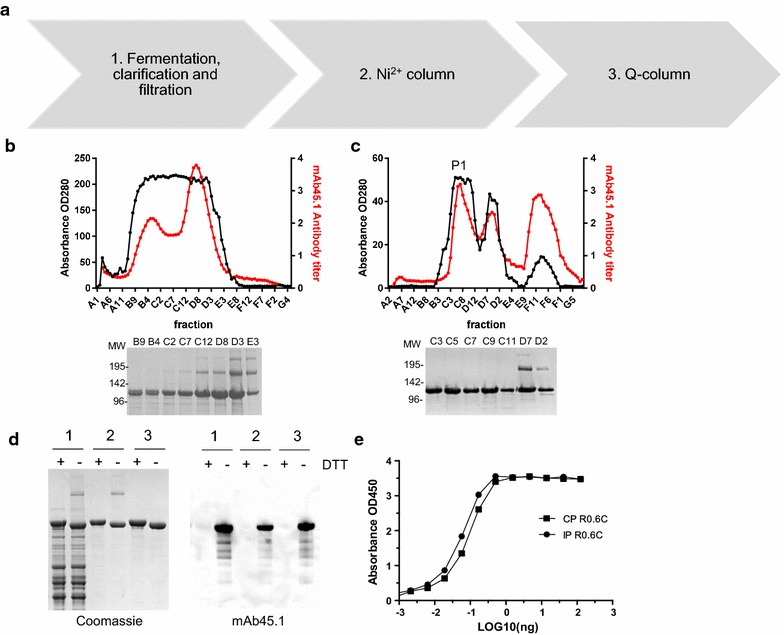
Expression and purification of R0.6C. **a** Schematic representation of expression and purification R0.6C fusion protein. Elution profiles of R0.6C on the **b** HisTrap HP and **c** HiTrap Q-HP-columns. *Red line* denotes UV absorbance (A280) and the *black line* denote antibody reactivity in the mAb45.1 sandwich ELISA. Selected fractions (5 µl) were analyzed on 4–12.5% polyacrylamide gels shown below the chromatograms. Protein was loaded without a reducing agent. The sizes (kDa) of the molecular mass markers are indicated. **d** Analysis of R0.6C. *Left panel* Coomassie blue-stained 4–12.5% polyacrylamide gel; *1* Supernatant, *2* HisTrap HP column and *3* HiTrap Q HP column purified R0.6C. *Right panel* an immune blot analysis of the same gel shown in the *left panel* using mAb45.1. Protein was loaded in each lane with (+) or without (−) DTT (10 mM). **e** Sandwich ELISA of conventionally (CP) and Immune (IP) purified R0.6C fusion proteins. Antigens were captured with mAb45.1 and detected with anti-His-HRP. *X-axis* is shown on a logarithmic scale

Since all constructs tested gave similar yields, we choose R0.6C with a C-terminal His-tag (Fig. 1a, construct no. 1) for optimization of fermentation in lab-scale bioreactors. The generation of R0.6C showed a substantial accumulation in the culture medium at 10–15 h post inoculation (Fig. 2a). Recombinant R0.6C was produced as an intact fusion protein as indicated by Coomassie staining (Fig. 2b upper panel) and immune blotting with an antibody against the C-terminal his-tag (Fig. 2b, middle panel). The secreted protein was properly folded as indicated by immune blotting with the conformation dependent mAb45.1 (Fig. 2b lower panel). Subsequently, a robust workflow for production was developed by growing *L. lactis* MG1363 expressing R0.6C in a 1 l stirred bioreactor for 15 h at 30 °C (Fig. 3a). The non-oxidative fermentation resulted in rapid acidification due to the production of lactate. Acidification eventually inhibits cell growth but also induces protein expression by activating the P170 promoter [18]. In order to optimize both cell growth and promoter activity, the fermenter was equipped with a pH electrode to monitor and control pH by addition of 2 M NaOH. The culture medium was also supplemented with 5 mM cysteine and 0.5 mM cystine which, together with the micro-aerobic milieu, is essential for high yield production of disulfide-bonded recombinant protein.

### Purification of recombinant R0.6C

Supernatants were concentrated and buffer exchanged for phosphate buffered saline (PBS) pH 7.4 supplemented with 15 mM imidazole. R0.6C was captured on a HisTrap HP column and bound protein was eluted with a linear imidazole gradient (Fig. 3b). Fractions containing recombinant R0.6C were analyzed by SDS-PAGE and by mAb45.1 sandwich ELISA (Fig. 3b). Fractions containing high concentration of immune reactive protein were pooled and loaded on an anion ion-exchange chromatography column, to separate protein species with native and non-native disulfide bonds (Fig. 3c). Fractions (P1) containing mAb45.1 reactive monomer were pooled with a major band of monomeric protein strongly reactive to mAb45.1 (Fig. 3d). This R0.6C fraction contained >80% properly folded *Pf*s48/45 relative to immune purified reference material (Fig. 3e and Additional file 1). The same work-flow for R0.6C constructs tagged with SpyTag, SpyCatcher, and mSA at their N-terminal ends resulted in similar yields of properly folded R0.6C.

### Mass spectrometry analysis of R0.6C

The mass of non-reduced and reduced full-length R0.6C was 71325.4 and 71331.3 Da as determined by LC–MS respectively (Additional file 2a, b). This molecular weight corresponds well to the predicted value of 71331.01 Da assuming that the fusion protein contains the vector-encoded amino acid residues A-E-R-S at the N-terminal end and a 6xHis-tag at the C-terminal end. Reduction of R0.6C resulted in a shift in the measured mass of the intact protein by approximately 6 Da, consistent with the presence of 3 disulfide bonds. The correct primary structure of R0.6C was further verified through MS/MS sequencing of 39 tryptic peptides derived from reduced R0.6C (Additional file 2c). These peptides cover 85% of the protein confirming that the purified R0.6C fusion protein was intact with the predicted amino acid residues corresponding to a full-length GLURP_27–500_–*Pf*s48/45_291–428_ fusion protein.

**Fig. 4:**
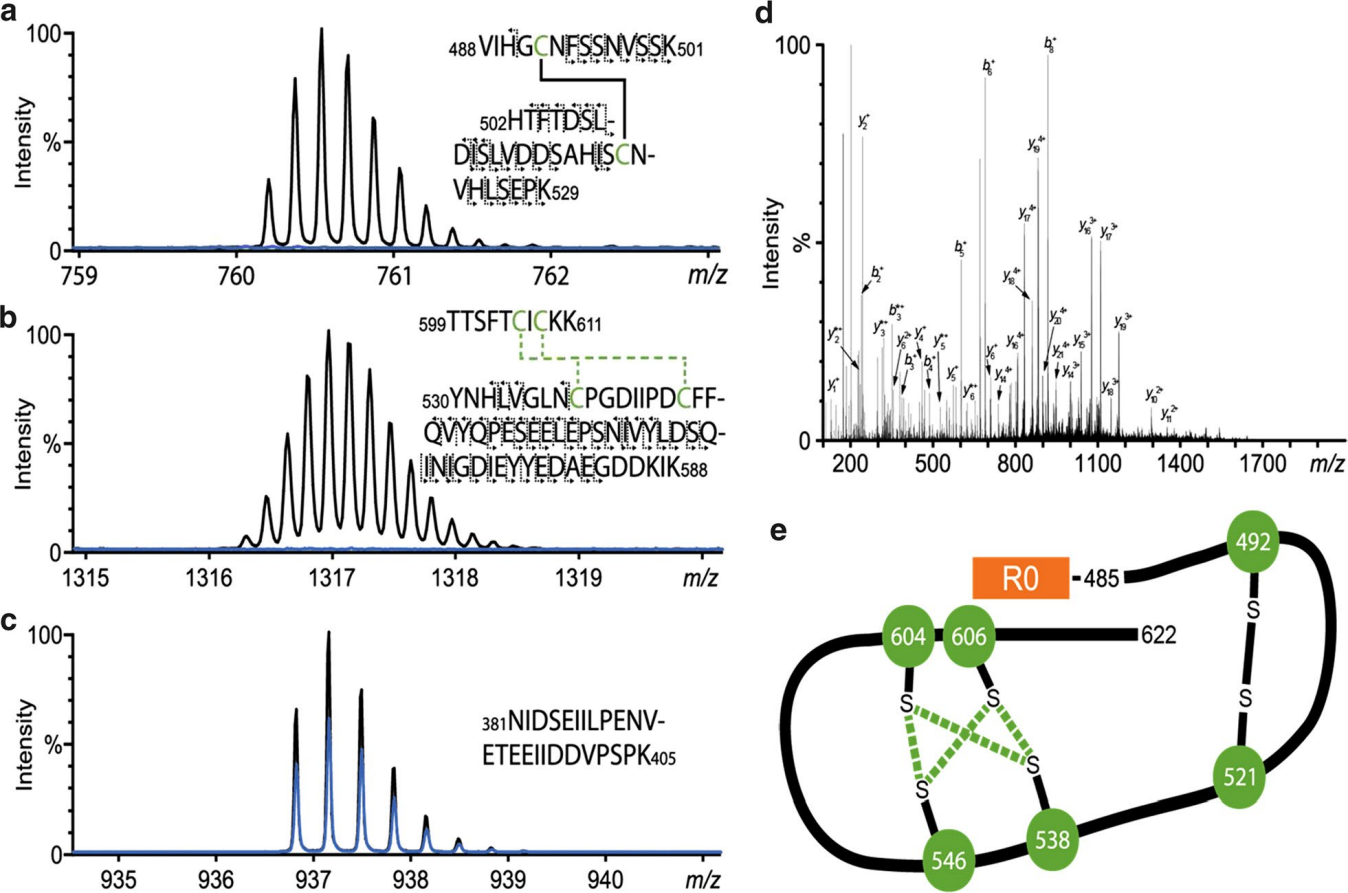
Mapping of disulphide-bonds in the *Pfs*48/45-6C region of R0.6C. R0.6C with and without reduction was digested with the protease trypsin prior to LC–MS/MS analysis. Two crosslinked peptides were identified and corresponding mass spectra of each peptide is shown in **a** and **b** with mass spectra corresponding to the non-reduced sample in *black* and the corresponding mass spectra from the reduced sample in *blue*. The proposed disulfide connectivity is indicated (*green dashed lines*) based on the extensive MS/MS fragmentation of each peptide (*solid black lines*). Several control peptides without cysteine residues were analyzed alongside to confirm equal sample load in each analysis, and spectra for one of these in shown **c**. Annotated MS/MS spectra of the peptide from **a** is shown in **d**. Schematic of the identified disulfide connectivity of *Pf*s48/45-6C in R0.6C is shown in **e**. The disulfide bonds that have been verified by LC–MS/MS is indicated by a *solid black line* and disulfide bonds indicated with *dashed green lines* is one of two possibilities

The disulfide connectivity of R0.6C was characterized by analyzing tryptic peptides by LC–MS/MS. Four peptides were not found in non-reduced R0.6C sample, indicating their involvement in cross-linking, whereas, two peptides were exclusively present in non-reduced R0.6C, referred to as XL-peptides I and II (Fig. 4a, b, respectively). The identity of these cross-linked peptides was established by manual inspection of masses of peptide precursors and the corresponding fragment ions (Fig. 4d for peptide I). In XL-peptide I, C492 formed a disulfide bond with C521. In XL-peptide II, C604 was disulfide bonded to C546 or C538, whereas C606 formed a disulfide bond with the other cysteine residue. Due to the close proximity of C604 and C606 it was only possible to resolve the disulfide connectivity to either of the two cysteine residues. Several control peptides without cysteine residues were monitored simultaneously with an example shown in (Fig. 4d). A diagram of the disulfide connectivity of the correctly folded *Pf*s48/45-6C region is shown in (Fig. 4e).

### Size-exclusion and reversed-phase HPLC analysis of purified R0.6C

**Fig. 5:**
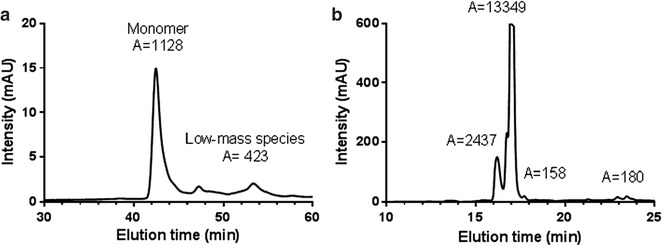
Size exclusion and reversed-phase HPLC analysis of purified R0.6C. **a** Size exclusion chromatography was performed under native conditions in a phosphate buffer pH 6.7, to determine the amount of monomer in the sample. The peak corresponding to the monomer is indicated with the integrated area of the peak written above. **b** Reversed-phase HPLC–UV chromatograms recorded following analysis of purified protein batches. The peak at 17 min corresponds to monomeric R0.6C antigen

A *Pf*s48/45-based vaccine antigen should consist of properly folded monomer. The final R0.6C batch elutes as a single peak at 42.5 min by analytical size exclusion chromatography (SEC), representing the active monomer state (Fig. 5a). Subsequent reversed-phase chromatography analysis showed a main peak with a retention time of 17.0 min and a smaller peak at approximately 16.5 min (Fig. 5b). The latter was absent in reference material containing immune-purified R0.6C (data not shown) suggesting that this peak corresponds to host cell proteins or a minor fraction of product-related impurities. The overall purity of R0.6C as determined by RP-HPLC was 87%.

### Stability of recombinant R0.6C

Finally, we assessed the chemical and physical stability of the recombinant protein for 30 days at 4 °C. There was no appreciable degradation at any time-point when analyzed by SDS-PAGE, immunoblotting with mAb45.1 (Additional file 3). The bioactivity of the protein was maintained throughout the experiment as determined by the sandwich-ELISA (Additional file 3).

### Immunogenicity of recombinant R0.6C

**Fig. 6:**
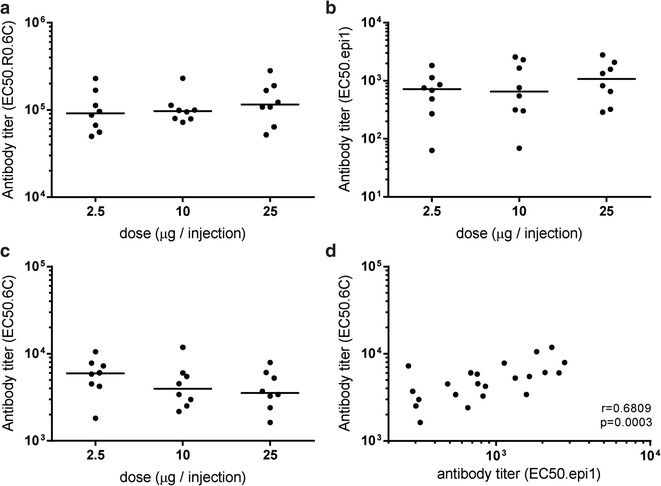
Immunogenicity of purified R0.6C. Rats were immunized with increasing doses (2.5, 10, and 25 µg) of purified R0.6C. Levels of specific antibodies were measured in the **a** R0.6C-ELISA, **b** mAb45.1 competitive ELISA, and **c** Pfs48/45-6C-ELISA. **e** Relationship between antibody levels determined in the 6C-ELISA and the 45.1 competition ELISA. Antibody titres are expressed as EC50 values.* The line* represents the median value. P values are based on the Mann–Whitney rank sum test

To ascertain that R0.6C elicit adequate levels of gametocyte-specific antibodies with the capacity to inhibit parasite fertilization, Wistar rats (N = 8) were immunized 3 times at 3-week intervals with increasing doses (2.5, 10, and 25 μg) of R0.6C inducing similar levels of vaccine-specific (Fig. 6a), epitope I-specific (Fig. 6b), and 6C-specific (Fig. 6c) antibodies. There was a strong correlation between 6C- and epitope I specific antibody levels (Fig. 6d) suggesting that epitope I is the main epitope in the 6-Cys domain of *Pf*s48/45. Collectively, these results demonstrate that R0.6C gives an adequate presentation of conformational epitope(s) in *Pf*s48/45. Serum pools from each dosing group were tested for functional activity in the SMFA showing >99% TB activity at a 1/9 dilution.

## Discussion

Production of recombinant protein is important for subunit vaccine development. Since there is no single universal host, which is perfect for production of all desired recombinant proteins, the selection of the right expression system is pivotal for development of a manufacturing process. The production host is not only important for the correct protein folding (protective antibodies often target conformational epitopes) but also process economics are key to keeping the price of the vaccine as low as possible, especially for vaccines targeting low-income countries.

From a manufacturing perspective, the major challenge with recombinant *Pf*s48/45 is production of correctly folded protein with sufficient yield (reviewed in [3]). One way to increase the amount of properly folded protein has been to produce *Pf*s48/45 in the *L. lactis* expression system genetically fused to the GLURP-R0 region [14, 15]. The efficient expression of disulfide-bonded protein in *L. lactis* was unexpected as this organism is low in its cysteine content and lacking known disulfide bond forming enzymes [26]. Thus, disulfide bond formation of recombinant *Pf*s48/45 most likely occurs spontaneously after secretion and is not only dependent on the primary structure but also on environmental factors that determine cysteine oxidation. By carefully optimizing the ratio of different redox couples in the fermentation broth, we have identified conditions, which render the extracellular milieu more oxidizing allowing formation of structural disulfide bonds in the *Pf*s48/45-6C domain of the fusion protein. These conditions seem to favor the formation of intramolecular disulfide bonds as secreted R0.6C was mainly present in the monomer state. However, the finding that crude R0.6C shows reduced reactivity with mAb45.1 compared to the reference material consisting of mAb45.1 immune-purified R0.6C [14] suggests that the monomer fraction contains a mixture of conformers with native and non-native cysteine connectivity. A work-flow has therefore been developed using batch fermentation in lab-scale stirred bioreactor to produce discreet batches of recombinant R0.6C purified by a simple 2-step purification process. The first step captures the recombinant protein from the culture supernatant and the second step aims to separate correctly folded and misfolded protein species. The SEC–HPLC analysis demonstrates that the final batch of R0.6C mainly contains monomers with all six cysteine residues in the oxidized state as shown by mass spectrometry analysis. The established disulfide connectivity of R0.6C is in excellent agreement with the disulfide bond connectivity of another recombinant protein containing the *Pf*s48/45-10C region (Mistarz et al., unpublished). The purity of the R0.6C batch is 87% as determined by RP-HPLC. Impurities in the final R0.6C batch are likely, at least in part, product-related consisting of misfolded protein species or degradation products.

Although the final yield of purified recombinant R0.6C is almost 25 mg per L culture broth, we suggest that further improvements are possible. Such improvements may include procedures to increase the biomass through systems where lactate is removed from the culture broth during fermentation using for example the REED™ (Reverse Electro-Enhanced Dialysis) technology [18]. Alternatively, product yield may be increased through adjusting the fermentation process, which is very easy to scale up to 200–1000 L since there is no requirement for oxygen and heavy stirring during fermentation. Finally, product yields can be increased through performing multiple fermentations that can be pooled before downstream purification.

Previously we have investigated the immunogenicity of *Pf*s48/45-based vaccine candidates using immune purified protein [14, 15, 27]. Here, we showed that *Pf*s48/45 fusion protein purified by conventional chromatographic procedures together with Montanide ISA 720 VG elicits TB antibodies at the lowest dose of 2.5 µg. Previously, doses below 4.7 µg failed to elicit TB antibodies when adjuvanted in Alhydrogel [14]. Improved formulations may contain immune modulators [28, 29] or may be based on novel delivery systems including virus like particles (VLP). This concept has been demonstrated recently for another TBV candidate, *Pf*s25, which was covalently coupled to VLPs [23, 24]. In this context, it is clearly important that the *L. lactis* expression system is able to accommodate a range of affinity tags added to either end of the recombinant protein.

In conclusion, we have demonstrated that *L. lactis* microbial factories are highly suited for the production of *Pf*s48/45. A manufacturing process for soluble and correctly folded R0.6C was developed and the resulting vaccine antigen induces high levels of functional antibodies. The high yield of correctly folded protein and the straightforward production process offers the possibility to investigate the vaccine potential of *Pf*s48/45 in human clinical trials.

## Methods

### Preparation of constructs

All the constructs are based on the *L. lactis* pSS1 plasmid vector [14]. The R0.6C construct with a C-terminal His tag has been described [14]. Non-tagged R0.6C was generated through PCR with forward primer (5′-GAAT GGA TCC TAC AAG TGA GAA TAG AAA TAA ACG) and reverse primer (5′-GAAT AGA TCT TTA TGC TGA ATC TAT AGT AAC TGT CAT ATA AGC) using R0.6C.6H gene as template. Codon optimized R0.6C.6H and N-terminal SpyCatcher and SpyTag [30] containing *BamH*I-*Bgl*II was synthesized by (GeneArt^®^ Life Technologies, Germany) and inserted into pSS1. For construction of chimeric N-terminal (mSA-R0.6C.6H) or C-terminal (R0.6C-mSA.6H) R0.6C, a synthetic DNA fragment encoding the monomeric Streptavidin (mSA) (GeneBank: 4JNJ_A) with a small Gly–Gly-Ser linker was synthesized by (GeneArt^®^ Life Technologies) and cloned into R0.6C.6H. The USP45-R0.6C.6H construct was generated by replacing the mutD310 signal peptide in pSS1 with the USP45 signal peptide [31] synthesized by (GeneArt^®^ Life Technologies) and cloned into R0.6C. All the constructs were verified by sequencing and subsequently transformed into *L. lactis* MG1363 by electroporation as described [32].

### Screening, fermentation and protein purification

*L. lactis* MG1363, containing R0.6C constructs was grown overnight at 30 °C in 5 ml LAB medium [15] supplemented with 4% glycerol–phosphate, 5% glucose and 5 µg/ml erythromycin. Culture supernatants were clarified by centrifugation at 9000*g* for 20 min and analysis of all the constructs was performed by Coomassie stained SDS-PAGE gel and Western blotting with mAb45.1 against conformational epitope I. Fermentation of *L. lactis* MG1363/R0.6C.6H was performed in a 1 l lab scale bioreactor at 30 °C with gentle stirring (150 rpm). For the time-course experiment 10 ml samples were withdrawn and used for analysis. Optical density at 600 nm (OD600) was used to assess cell density. Cell-free culture-filtrates were concentrated tenfold and buffer exchanged into phosphate buffered saline (PBS) pH 7.4 supplemented with 15 mM imidazole) using a QuixStand Benchtop system (Hollow fiber cartridge with cutoff at 50,000 Da, surface area 650 cm^2^, GE Healthcare, Sweden) followed by filtration through a Durapore filter (PVDF, 0.22 µm, Millipore) and applied to a 5 ml HisTrap HP column (GE Healthcare, Sweden). Bound protein was step gradient eluted with 500 mM imidazole in tris buffer pH 8.0 (55 mM tris, 21 mM NaCl) at a flow rate of 4 ml/min and fractions containing the desired protein were applied to a 5 ml HiTrap Q HP column (GE Healthcare, Sweden). Bound protein was eluted through step gradient elution in tris buffer pH 8.0 (55 mM tris, 1 M NaCl) and fractions containing monomers with the highest amount of mAb45.1-reactive protein were concentrated by a VIVA spin column 30 kDa cutoff (GE Healthcare, Sweden), and kept in 55 mM tris, 300 mM NaCl, 0.025% Tween 80 and 1 mM EDTA, pH 8.0 at −80 °C until use. Immune purification of R0.6C was performed as described earlier [14, 15]. Analysis of all the fractions was performed by SDS-PAGE. Immunoblotting was performed with mAb45.1 against conformational epitope I. Protein concentration was measured using either the BCA protein assay (Thermo Fisher Scientific) or by densitometric analysis of Coomassie stained SDS-PAGE gel using Image Quant TL (IQTL) software (GE Healthcare).

### SEC–HPLC and RP-HPLC analysis of R0.6C

Native size exclusion high-performance liquid chromatography (SEC–HPLC) or reversed-phase HPLC (RP-HPLC) of intact R0.6C were performed using an Agilent 1100 Series HPLC System (Agilent Technologies, USA) equipped with a TSKgel G3000SWXL SEC column, 5 µm, 7.8 × 300 mm (Tosoh Bioscience, Japan) or equipped with a Vydac 214TP C4 reversed-phase column, 5 µm, 4.6 × 250 mm (The Separations Group, CA, US), respectively. 210 pmol of protein was loaded on the SEC column and eluted with a 0.15 ml/min flow of elution buffer (200 mM phosphate, 0.65 g/l l-arginine, 0.05% NaN_3_, pH 6.7) at room temperature. For RP-HPLC, 210 pmol of protein was loaded on the RP-column and eluted with a linear gradient of 3–95% of 0.1% trifluoroacetic acid (TFA), 20% isopropanol and 70% acetonitrile over 30 min. The absorbance was measured at 280 or 215 nm for SEC–HPLC or RP-HPLC, respectively, and chromatographic peaks were integrated by HPLC ChemStation (Agilent Technologies, CA, US).

### Mass spectrometry

Accurate molecular mass of full-length R0.6C was measured by LC–ESI–MS. 30 pmol of protein were loaded on a C4 pre-column (Acquity UPLC Protein BEH C4 Vanguard, 1.7, 2.1 × 5 mm, Waters, USA) and eluted onto a Q-TOF mass spectrometer (Synapt G2 HDMS, Waters, UK) with a chromatographic gradient. LC–MS data where recorded and analyzed by MassLynx software (Waters, UK). Mass spectra were deconvoluted using the MaxEnt 1 algorithm in the MassLynx software. Peptide mapping of R0.6C was performed by LC–MS/MS of tryptic digests. Guanidine hydrochloride, dithiothreitol and iodoacetamide were added to 100 pmol of protein. This was followed by addition of iodoacetamide and the protease trypsin. Tryptic peptides were desalted and chromatographically separated over 40 min on a UHPLC C18 pre column and column, respectively (Acquity UPLC BEH column, Waters, USA) using a nanoACQUITY UPLC system (Waters, USA). The eluate ionized by electrospray ionization and data were acquired on the same mass spectrometer software as described above. LC–MS/MS data were analyzed using PLGS (waters, USA) and peptide maps were created using DynamX (Waters, USA). Cysteine connectivity characterization was done with identical UPLC conditions as for peptide mapping. Tryptic digestion was additionally performed under similar conditions, with and without addition of guanidine hydrochloride, dithiothreitol or iodoacetamide to produce peptides without and without intact cysteine bonds, respectively. Collision induced dissociation (CID) fragmentation was performed on the two identified cross-linked peptides to produce MS/MS spectra used for manual analyzing the MS/MS fragments by MassLynx software for characterization of cysteine connectivity.

### Animals and immunogenicity studies

Female Wistar Hannover rats (Taconic, Denmark) and kept in the Laboratory Animal Facility Centre at Panum, University of Copenhagen, Denmark for 7 days before the first immunization. All procedures regarding animal immunizations complied with European and National regulations. Groups of eight rats were immunized by the s.c. route 3 times at 3-week intervals with increasing doses (2.5, 10, and 25 μg) of R0.6C. Protein preparations were emulsified in Montanide ISA720 VG (Seppic) immediately before use. Responses were measured using sera taken three weeks after the third immunization (Day 63).

### Enzyme-linked immunosorbent assay (ELISA)

The mAb45.1 sandwich ELISA was performed as previously described [14, 15]. The coating concentration of recombinant *Pf*s48/45 (6C) and R0.6C was 0.5 µg/ml and bound antibodies were detected with HRP-conjugated rabbit-anti rat IgG-HRP (DAKO, Denmark) as described in detail [27]. The mAb45.1 competition ELISA was performed as follows; Briefly, ELISA MaxiSorp microtitre plates (Sterilin^®^ ELISA plates, The Netherlands) were coated at 4 °C overnight with 100 µl per well of 5 µg/ml of mAb45.1 in PBS pH 7.4. Wells were blocked with 150 µl of 5% non-fat skimmed milk powder in PBS for 1 h followed by three washings with PBS containing 0.05% Tween 20 (PBST). Sera at threefold dilutions (50 µl) were mixed with 50 µl R0.6C (1 µg/ml) in PBST, added to the wells and incubated for 4 h at room temperature. Wells were washed with PBST and bound antigen was revealed with anti-His-HRP (1/6000 in PBST) for 2 h at room temperature. Midpoint (EC50) values were calculated using GraphPad Prism 7, (GraphPad Software, USA).

### Standard membrane feeding assay (SMFA)

The biological activity of anti-R0.6C antibodies was assessed in the SMFA as previously described in detail [15]. Estimates of transmission reducing activity (TRA) were generated using oocyst densities in individual mosquitoes, comparing test feeders with control feeders from the same experiment as described [33].

### Statistical analysis

The Mann–Whitney rank sum test was used for analyzing differences between groups. P < 0.05 was considered to be statistically significant.

## Additional files

**Additional file 1.** Production and purification of R0.6C.

**Additional file 2.** Intact mass analysis of R0.6C. Mass spectra of full-length antigens by LC–MS and peptide maps from LC–MS/MS of trypsin digested antigens. (a) Deconvoluted mass spectra of non-reduced R0.6C. (b) Deconvoluted mass spectra of reduced R0.6C. (c)Tryptic peptide maps of R0.6C. In brackets is the calculated accuracy of the measurement to the predicted mass in ppm. All measurements were done in duplicates.

**Additional file 3.** Stability of R0.6C. R0.6C was incubated at 4 °C for 0, 15 and 30 days. Three and 1 µg of R0.6C with (+) and without (−) DTT was analyzed by (a) SDS-PAGE coomassie staining, (b) Immune-blotting using mAb 45.1 and (c) Sandwich ELISA. Antigens were captured with mAb45.1 and detected with anti-His-HRP.

## Abbreviations

6C: 6-Cys-region of the *Plasmodium falciparum* s48/45 protein
DTT: dithiothreitol
GLURP: glutamate-rich protein of *Plasmodium falciparum*
MSP3: merozoite surface protein 3 of *Plasmodium falciparum*
IEC: ion exchange chromatography
IMAC: immobilized metal ion chromatography
LC–MS: liquid chromatography coupled with mass spectrometry
LC–MS/MS: liquid chromatography coupled with tandem mass spectrometry
mAb45.1: antibody binding 45.1 (epitope I) of the *Plasmodium falciparum* 48/45 protein
*Pf*s48/45: *Plasmodium falciparum* s48/45 protein
R0: r0-region of the glutamate-rich protein (GLURP)
SEC: analytical size-exclusion chromatography of intact proteins under native conditions
SMFA: standard membrane feeding assay
TB: transmission-blocking
XL: cross-linked peptide
