# Supplementary material for: Construct design, production, and characterization of Plasmodium falciparum 48/45 R0.6C subunit protein produced in Lactococcus lactis as candidate vaccine
Source: Microb Cell Fact. 2017 May 31;16:97. doi: 10.1186/s12934-017-0710-0 (PMC5452637; doi:10.1186/s12934-017-0710-0)
Supplement: Supplementary file 1 — Additional file 1. Production and purification of R0.6C. [file 12934_2017_710_MOESM1_ESM.docx]

**Additional file 1:** Production and purification of R0.6C.

|  |  | R0.6C | | |
| --- | --- | --- | --- | --- |
|  | Yield^a^  (mg/L) | Monomer^b^  (%) | Purity^c^  (%) | Folding^d^  (%) |
| Supernatant | 45 | ND | ND | ND |
| HisTrap HP | 40 | >90 | ND | 65 |
| HiTrap Q-HP (CP) | 25 | >99 | >87 | >80 |

^a^ Yield is determined by BCA assay as well as inspection of Coomassie blue stained SDS-PAGE under reducing (10 mM DTT) and or non-reducing conditions using ImageQuantTL 8.1 Software (GE Healthcare).

^b^ Monomer is given as (amount of total R0.6C monomer/ amount of total R0.6C multimer) X 100 % determined by SE-HPLC.

^c^ Purity of CP R0.6C as determined by RP-HPLC. % of purity was calculated using formula (amount of total R0.6C monomer / amount of total protein) X 100 %.

^d^ Immune-purified R0.6C used as a reference for calculation of correctly folded R0.6C.
