# Supplementary material for: Construct design, production, and characterization of Plasmodium falciparum 48/45 R0.6C subunit protein produced in Lactococcus lactis as candidate vaccine
Source: Microb Cell Fact. 2017 May 31;16:97. doi: 10.1186/s12934-017-0710-0 (PMC5452637; doi:10.1186/s12934-017-0710-0)
Supplement: Supplementary file 2 — Additional file 2. Intact mass analysis of R0.6C. Mass spectra of full-length antigens by LC–MS and peptide maps from LC–MS/MS of trypsin digested antigens. (a) Deconvoluted mass spectra of non-reduced R0.6C. (b) Deconvoluted mass spectra of reduced R0.6C. (c)Tryptic peptide maps of R0.6C. In brackets is the calculated accuracy of the measurement to the predicted mass in ppm. All measurements were done in duplicates. [file 12934_2017_710_MOESM2_ESM.pptx]

## Slide 1
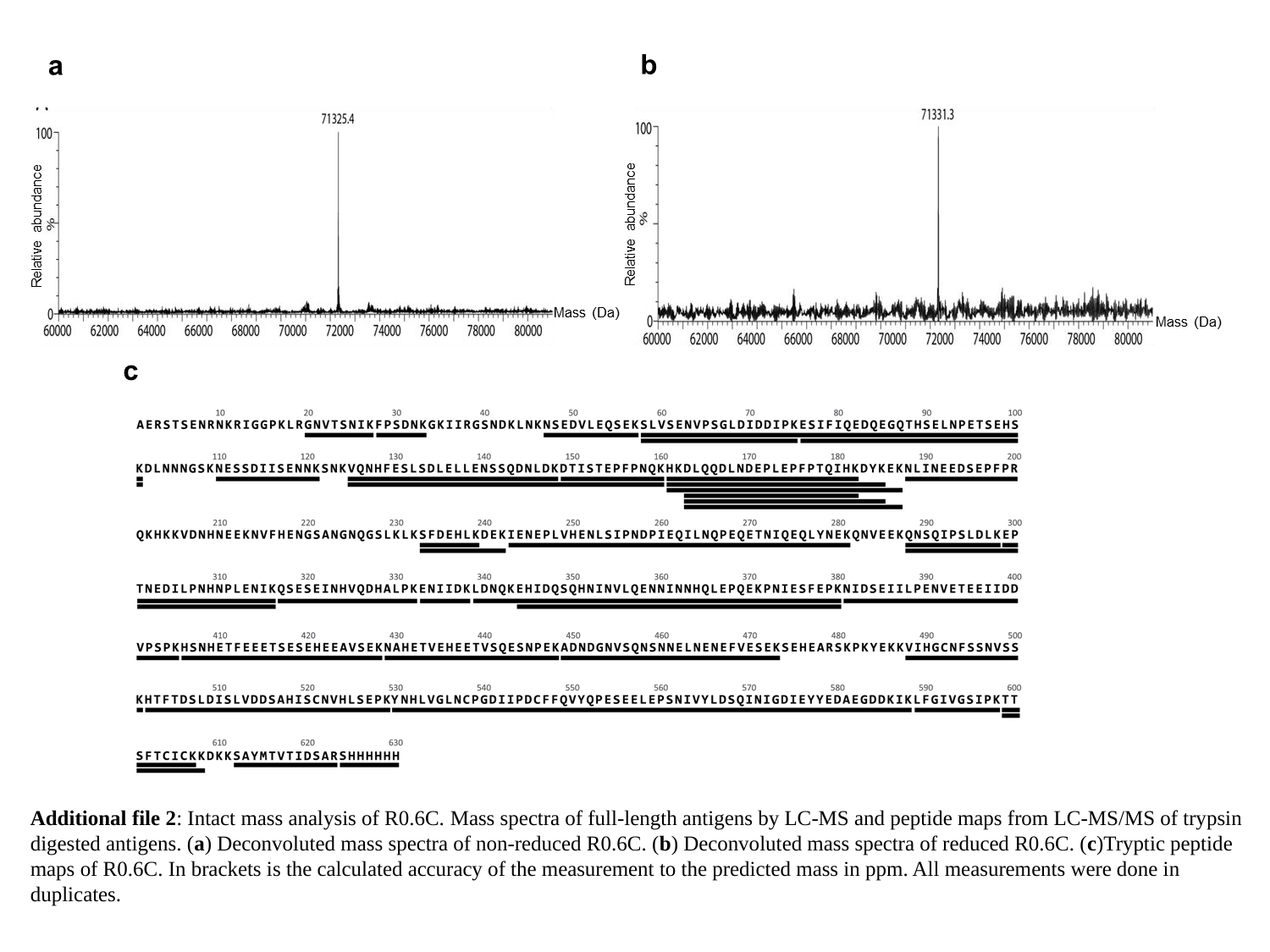

Additional file 2: Intact mass analysis of R0.6C. Mass spectra of full-length antigens by LC-MS and peptide maps from LC-MS/MS of trypsin digested antigens. (a) Deconvoluted mass spectra of non-reduced R0.6C. (b) Deconvoluted mass spectra of reduced R0.6C. (c)Tryptic peptide maps of R0.6C. In brackets is the calculated accuracy of the measurement to the predicted mass in ppm. All measurements were done in duplicates.
