# Supplementary material for: Construct design, production, and characterization of Plasmodium falciparum 48/45 R0.6C subunit protein produced in Lactococcus lactis as candidate vaccine
Source: Microb Cell Fact. 2017 May 31;16:97. doi: 10.1186/s12934-017-0710-0 (PMC5452637; doi:10.1186/s12934-017-0710-0)
Supplement: Supplementary file 3 — Additional file 3. Stability of R0.6C. R0.6C was incubated at 4 °C for 0, 15 and 30 days. Three and 1 µg of R0.6C with (+) and without (−) DTT was analyzed by (a) SDS-PAGE coomassie staining, (b) Immune-blotting using mAb 45.1 and (c) Sandwich ELISA. Antigens were captured with mAb45.1 and detected with anti-His-HRP. [file 12934_2017_710_MOESM3_ESM.pptx]

## Slide 1
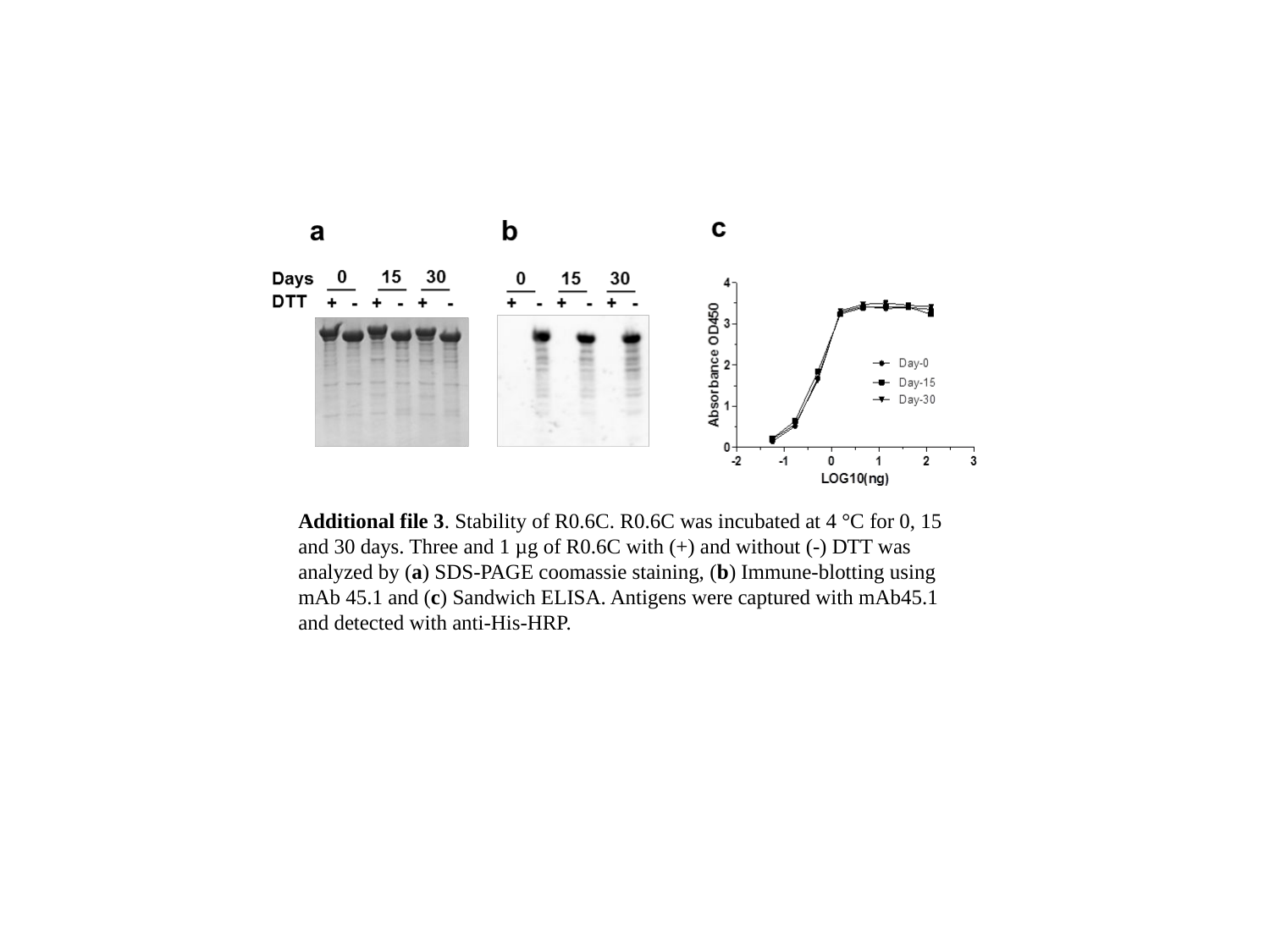

Additional file 3. Stability of R0.6C. R0.6C was incubated at 4 °C for 0, 15 and 30 days. Three and 1 µg of R0.6C with (+) and without (-) DTT was analyzed by (a) SDS-PAGE coomassie staining, (b) Immune-blotting using mAb 45.1 and (c) Sandwich ELISA. Antigens were captured with mAb45.1 and detected with anti-His-HRP.
